# Safety and effectiveness of nivolumab in Japanese patients with malignant melanoma: Final analysis of a post‐marketing surveillance

**DOI:** 10.1111/1346-8138.16432

**Published:** 2022-05-23

**Authors:** Hisashi Uhara, Tetsuya Tsuchida, Yoshio Kiyohara, Ayumi Akamatsu, Takahiko Sakamoto, Naoya Yamazaki

**Affiliations:** ^1^ Department of Dermatology Sapporo Medical University School of Medicine Sapporo Japan; ^2^ Department of Dermatology Saitama Medical University Saitama Japan; ^3^ Dermatology Division Shizuoka Cancer Center Hospital Shizuoka Japan; ^4^ Pharmacovigilance Division Ono Pharmaceutical Osaka Japan; ^5^ Department of Dermatologic Oncology National Cancer Center Hospital Tokyo Japan

**Keywords:** Japan, malignant melanoma, nivolumab, post‐marketing surveillance, real‐world

## Abstract

Nivolumab, a monoclonal antibody against human programmed death 1, was approved for the treatment of melanoma in July 2014 in Japan. Because the Japanese phase II studies (ONO‐4538‐02, ONO‐4538‐08) enrolled small numbers of melanoma patients, post‐marketing surveillance (PMS; JapicCTI‐163 272) was conducted to collect safety data in a larger patient population. We report data for melanoma patients who received nivolumab between July 4, 2014 and February 28, 2017. Data collected included baseline characteristics, laboratory tests, treatment‐related adverse events (TRAE), and overall survival (OS). Of 2069 enrolled patients, 2008 patients were included in the safety analysis population. There were 1030 (51.3%) males, the median age was 69 years, and 269 patients (13.4%) had a performance status of ≥2. The primary tumor sites were cutaneous (34.4%), mucosal (34.2%), acral lentiginous (18.6%), others (6.8%), and unknown (6.3%). TRAE occurred in 62.1% of patients, the most common being hypothyroidism (14.0%), increased aspartate aminotransferase (8.5%), and increased alanine aminotransferase (6.9%). TRAE of special interest in ≥5% of patients were thyroid dysfunction (24.9%), hepatic dysfunction (20.6%), infusion reactions (11.4%), colitis/severe diarrhea (6.3%), and interstitial lung disease (ILD; 5.0%). Several types of TRAE of special interest, which included myasthenia gravis/myocarditis/myositis/rhabdomyolysis (0.9%), venous thromboembolism (0.2%), immune thrombocytopenic purpura (0.1%), and encephalitis (0.0%), were observed in this PMS. Although these TRAE were not reported in previous studies (ONO‐4538‐02, ONO‐4538‐08, CheckMate 066, and CheckMate 037), they have been listed in the current Risk Management Plan. History of ILD and male sex were risk factors for ILD in a multivariable analysis. Age <75 years was a risk factor for hepatic dysfunction. At 12 months, median OS was not reached. In conclusion, these results suggested that there was no concern requiring additional precautions for the safety of nivolumab in Japanese patients with melanoma other than the safety information in the Risk Management Plan.

## 1 INTRODUCTION

Melanoma is a relatively rare malignancy in Japan with an estimated incidence of only 1.75/100 000 people.^1^ The percentages of patients with acral lentiginous melanoma (40.4%), superficial spreading melanoma (20.5%), nodular melanoma (10.0%), lentiginous melanoma (8.1%), and mucosal melanoma (9.5%)^2^ differ somewhat from those reported in Western countries (4.6%, 54.1%, 13.8%, 3.9%, and 0.4%, respectively).^3^ In particular, the proportions of patients with acral lentiginous or mucosal melanoma are higher in Japan than in Western countries. Moreover, these characteristics appear to influence the distribution of clinical stage at the initial diagnosis because melanoma is usually diagnosed at more advanced stages in Japanese than in Europeans and Americans (Japanese vs Europeans and Americans: stage III, 24.1% vs 7.5%; stage IV, 6.2% vs 2.5%).^2^, ^4^

Nivolumab was the world's first humanized monoclonal antibody against human programmed death 1 (PD‐1). Nivolumab is an immune checkpoint inhibitor with anticancer activities that restores the immune function of cytotoxic T lymphocytes, which eliminate tumor cells.^5^, ^6^ In July 2014, nivolumab was approved in Japan at a dose of 2 mg/kg every 3 weeks (Q3W) for advanced melanoma patients who had previously received chemotherapy, based on the results of a phase II study (ONO‐4538‐02).^7^ This approval was extended in February 2016 to include 3 mg/kg Q2W as first‐ or second‐line therapy, following the ONO‐4538‐08,^8^ CheckMate 037,^9^ and CheckMate 066^10^ studies. However, those clinical studies excluded patients with an Eastern Cooperative Oncology Group (ECOG) performance status (PS) ≥2 or other clinical factors. In addition, relatively few patients were enrolled in the Japanese clinical studies: 35 in ONO‐4538‐02^7^ and 24 in ONO‐4538‐08.^8^ Therefore, post‐marketing surveillance (PMS) of nivolumab in patients with malignant melanoma was conducted in Japan to collect safety data in a larger patient population. The PMS is a Japan‐specific system, and the Ministry of Health, Labor and Welfare requested this PMS as part of the approval condition for nivolumab. Here, we report the results for all patients registered up to February 28, 2017 as the final analysis of the PMS.

## 2 METHODS

### 2.1 Survey design and patients

This prospective, non‐interventional, observational PMS evaluated the safety and effectiveness of nivolumab for 12 months after the first dose in Japanese patients with malignant melanoma. This PMS conformed to Japanese Good Post‐Marketing Study Practice regulations. Each participating hospital agreed to contracts for this surveillance with the study sponsor. No intervention was performed for the purpose of this study. This PMS was registered at clinicaltrials.jp (JapicCTI‐163272).

In line with the approved Japanese label, patients with unresectable malignant melanoma are eligible for nivolumab. From the approval date of July 4, 2014, all patients with malignant melanoma who were planned to receive nivolumab were to be registered. Although registration was planned to continue until May 2021, we only collected the case report forms for patients registered through to February 28, 2017, because the planned number of cases^11^ had been reached. In this report, we assessed the case report forms for patients treated at 342 hospitals, which permitted data publication.

Patients intravenously received a nivolumab dose of 2 mg/kg Q3W or 3 mg/kg Q2W as second‐/later‐line therapy, and 3 mg/kg Q2W as first‐line therapy. Then, they were monitored for 12 months after the first dose. Patients who discontinued nivolumab treatment within 12 months were also followed up, if possible, for 12 months after the first nivolumab dose.

### 2.2 Data collection

We collected patients' baseline demographic characteristics, including age, sex, weight, ECOG PS, smoking and alcohol consumption history, medical history, cancer stage, primary tumor site, and previous treatments (e.g., surgery and radiotherapy). The duration of nivolumab administration, the number of doses, and the reason for discontinuation were also recorded. Data on laboratory tests, particularly aspartate transaminase, alanine transaminase, γ‐glutamyltransferase, alanine phosphatase, bilirubin, thyroid‐stimulating hormone, free triiodothyronine (FT3), and free thyroxine (FT4), were regularly recorded.

The primary outcome was the incidence of treatment‐related adverse events (TRAE). We used the Japanese version of the Medical Dictionary for Regulatory Activities version 23.0 and the National Cancer Institute Common Terminology Criteria for Adverse Events version 4.0 to classify and grade TRAE, respectively. Furthermore, we evaluated the incidences of TRAE among patients stratified by baseline factors.

The Risk Management Plan for nivolumab defined the following TRAE categories of special interest: interstitial lung disease (ILD); myasthenia gravis/myocarditis/myositis/rhabdomyolysis; colitis and severe diarrhea; type 1 diabetes mellitus; hepatic dysfunction; thyroid dysfunction; neurological disorder; renal disorders; adrenal disorders; encephalitis; severe skin disorders; venous thromboembolism; infusion reaction; immune thrombocytopenic purpura; and cardiac disorders, such as atrial fibrillation, bradycardia, and ventricular extrasystole.

We also evaluated the effectiveness of nivolumab in terms of the estimated median overall survival (OS).

### 2.3 Data analyses

Safety data were evaluated for all patients, after excluding any patients in whom nivolumab was not used and duplicated patients. The effectiveness analysis set comprised all patients in the safety analysis population, excluding those treated with nivolumab for an off‐label purpose.

The frequencies of TRAE were compared among subgroups of patients using Fisher's exact test, Wilcoxon's rank‐sum test, or the χ^2^‐test, as appropriate, with cross‐tabulations with other items for factors showing significant differences among subgroups.

To identify patient characteristics that may affect the development of ILD or hepatic dysfunction, competing risk analyses were performed using the Fine and Gray proportional subdistribution hazards model.^12^

Overall survival was defined as the number of days from the start of nivolumab treatment to death, and survival curves for all patients were plotted using the Kaplan–Meier method. Patients were censored if the outcome was unknown, lost to follow‐up, or transferred to another hospital. In these circumstances, the date of the last observation was used as the censor date.

SAS statistical software (SAS Institute Japan Ltd.) version 9.4 TS1M4 was used for all statistical analyses.

## 3 RESULTS

### 3.1 Patients

**FIGURE 1:**
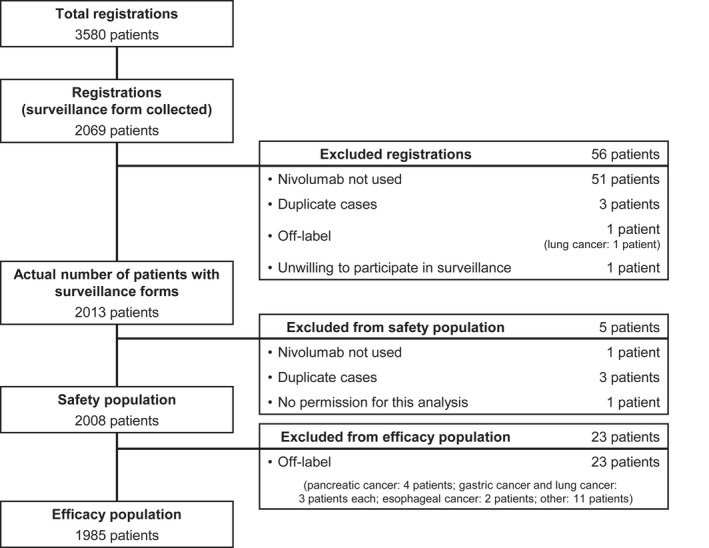
Patient disposition

A total of 2069 patients were registered and case report forms collected between July 4, 2014 and February 28, 2017 (Figure 1). Of these, 2008 were included in the safety analysis population, and 1985 were included in the effectiveness analysis population after excluding 23 patients who were treated with nivolumab for off‐label purposes. Among the 2008 patients in the safety population, 1030 (51.3%) were male and 978 (48.7%) were female. The median age was 69 years (range, 15–94) and 638 (31.8%) were ≥75 years old (Table 1). The PS was ≥2 in 269 patients (13.4%), which included 95 (4.7%) with a PS of 3 and 29 (1.4%) with a PS of 4.

**TABLE 1:** Baseline patient characteristics

Characteristic	Category	Value
N		2008
Sex	Male	1030 (51.3)
	Female	978 (48.7)
Age (years)	Median (range)	69 (15–94)
Age groups	<15	0
	≥15 to <65	724 (36.1)
	≥65 to <75	646 (32.2)
	≥75	638 (31.8)
ECOG PS	0	1223 (60.9)
	1	515 (25.6)
	2	145 (7.2)
	3	95 (4.7)
	4	29 (1.4)
	Unknown	1 (0.0)
Medical history	Yes	1362 (67.8)
	Lung	164 (8.2)
	Liver	181 (9.0)
	Kidney	94 (4.7)
	Thyroid	153 (7.6)
	Autoimmune disease	65 (3.2)
	No	635 (31.6)
	Unknown	11 (0.5)
New onset/recurrence	New onset	897 (44.7)
	Recurrence	1068 (53.2)
	Unknown	43 (2.1)
Primary site^a^	Cutaneous melanoma	691 (34.4)
	Mucosal melanoma (including eye)	686 (34.2)
	Acral lentiginous melanoma^b^	374 (18.6)
	Other	136 (6.8)
	Unknown	127 (6.3)
Stage	III	222 (11.1)
	IV	1672 (83.3)
	Other	39 (1.9)
	Unknown	75 (3.7)
Metastasis	No	88 (4.4)
	Yes	1894 (94.3)
	Unknown	26 (1.3)
	Region	
	Brain	222 (11.1)
	Lung	882 (43.9)
	Liver	614 (30.6)
	Lymph node	1195 (59.5)
	Bone	473 (23.6)
	Other	635 (31.6)
	Unknown	1 (0.0)

*Note*: Values are n (%) unless otherwise specified.

Abbreviation: ECOG PS, Eastern Cooperative Oncology Group performance status.

^a^ Six patients had multiple primary tumor sites; the total number of patients was counted.

^b^ Patients whose primary tumor site was recorded as cutaneous melanoma and acral lentiginous melanoma were classified into acral lentiginous melanoma.

The primary tumor sites were cutaneous melanoma in 691 patients (34.4%), mucosal melanoma in 686 patients (34.2%), acral lentiginous melanoma in 374 patients (18.6%), others in 136 (6.8%) patients, and unknown in 127 patients (6.3%). Six patients had multiple primary tumor sites. There were 897 patients (44.7%) with newly diagnosed melanoma and 1068 (53.2%) with recurrent melanoma.

### 3.2 Treatment

Nivolumab was administered as first‐line therapy in 691 patients (34.4%) and as second‐/later‐line in 1187 (59.1%). The median number of nivolumab doses was 7 (range, 1–36). Overall, 722 patients (36.0%) had received <4 doses (Table S1). Nivolumab was administered at a dose of 2 mg/kg Q3W in 1458 patients (72.6%) and at a dose of 3 mg/kg Q2W in 433 patients (21.6%). A total of 428 patients (21.3%) were still receiving nivolumab at 12 months. The other 1580 patients (78.7%) stopped treatment during the observation period for reasons that included lack of effectiveness in 933 (46.5%), adverse events in 304 (15.1%), disease progression in 143 (7.1%), and death in 134 (6.7%) (Table 2).

**TABLE 2:** Reasons for treatment discontinuation

	n (%)
Discontinued treatment	1580 (78.7)
Reasons for treatment discontinuation	
Responded to nivolumab	31 (1.5)
Insufficient effectiveness	933 (46.5)
Adverse event	304 (15.1)
Transferred hospital	65 (3.2)
Death	134 (6.7)
Disease progression	143 (7.1)
Other	81 (4.0)

### 3.3 Safety

#### 3.3.1 Overall safety

**FIGURE 2:**
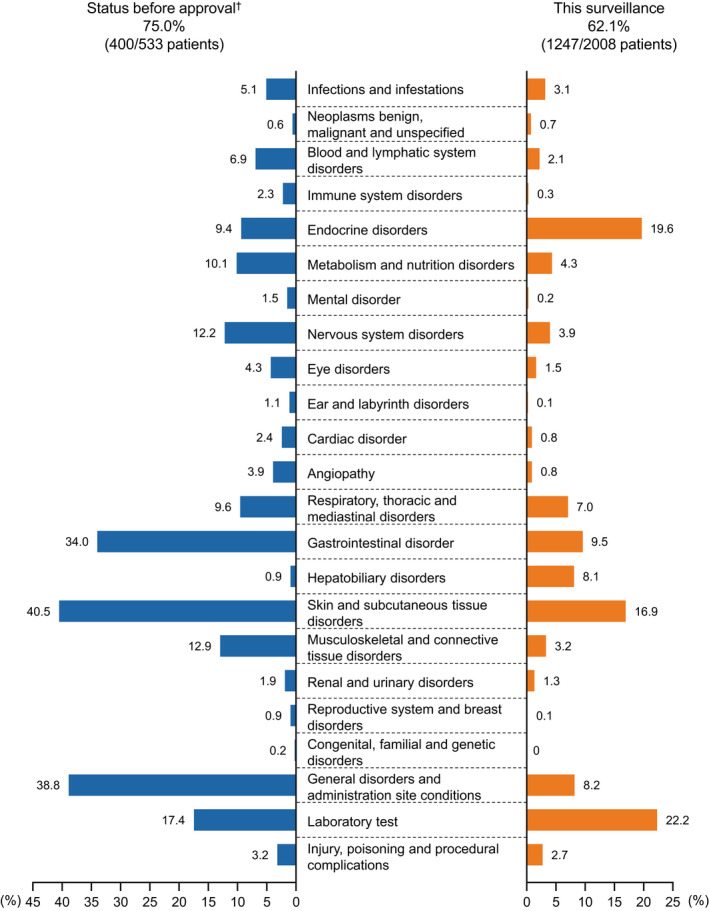
Treatment‐related adverse events by system organ class. The types of treatment‐related adverse events were classified using MedDRA/J Ver. 23.0, by system organ class/preferred term. ^†^Data derived from Japanese phase II studies (ONO‐4538‐02, ONO‐4538‐08)^7^, ^8^ and global phase III studies (CheckMate 066, CheckMate 037)^9^, ^10^.

Treatment‐related adverse events occurred in 1247 patients (62.1%), including grade ≥3 TRAE in 394 (19.6%). The three most common TRAE were hypothyroidism (14.0%), increased aspartate aminotransferase (8.5%), and increased alanine aminotransferase (6.9%) (Figure 2, Tables S2 and S3).

Possible relationships between death and nivolumab treatment could not be ruled out in 43 patients. The TRAE leading to death included “no other adverse events related to death” (11 patients), ILD (seven patients), sepsis (four patients), hepatic function abnormal (three patients), disseminated intravascular coagulation (three patients), respiratory failure (two patients), hepatic failure (two patients), acute kidney injury (two patients), and *Pneumocystis jirovecii* pneumonia (two patients).

#### 3.3.2 Safety in subgroups of patients

The frequencies of TRAE differed significantly among patients divided by baseline factors such as PS, medical history, history of thyroid disease, and number of doses of nivolumab (Tables 3 and S4).

**TABLE 3:** Treatment‐related adverse events by baseline factors

Baseline factors		Patients	Patients experiencing any TRAE			
		n (%)	n (%)	95% CI of incidence rate	*p* ^†^	Test
All		2008	1247 (62.1)	59.9–64.2		
Sex	Male	1030 (51.3)	645 (62.6)	59.6–65.6	0.6454	F
	Female	978 (48.7)	602 (61.6)	58.4–64.6		
Age (years)	<15	0	0	ND	0.1868	W
	≥15 to <65	724 (36.1)	462 (63.8)	60.2–67.3		
	≥65 to <75	646 (32.2)	400 (61.9)	58.0–65.7		
	≥75	638 (31.8)	385 (60.3)	56.4–64.2		
ECOG PS	0–1	1738 (86.6)	1123 (64.6)	62.3–66.9	**<0.0001**	W
	2–4	269 (13.4)	123 (45.7)	39.7–51.9		
	Unknown	1 (0.0)	1 (100.0)	ND		
BMI (kg/m^2^)	<18.5	264 (13.1)	150 (56.8)	50.6–62.9	**0.0207**	W
	≥18.5 to <25	1257 (62.6)	783 (62.3)	59.5–65.0		
	≥25	458 (22.8)	301 (65.7)	61.2–70.1		
	Unknown	29 (1.4)	13 (44.8)	ND		
Medical history	No	635 (31.6)	356 (56.1)	52.1–60.0	**0.0002**	F
	Yes	1362 (67.8)	885 (65.0)	62.4–67.5		
	Unknown	11 (0.5)	6 (54.5)	ND		
History of liver disease	No	1822 (90.7)	1124 (61.7)	59.4–63.9	0.2974	F
	Yes	181 (9.0)	119 (65.7)	58.3–72.6		
	Unknown	5 (0.2)	4 (80.0)	ND		
History of kidney disease	No	1907 (95.0)	1175 (61.6)	59.4–63.8	0.1028	F
	Yes	94 (4.7)	66 (70.2)	59.9–79.2		
	Unknown	7 (0.3)	6 (85.7)	ND		
History of lung disease	No	1840 (91.6)	1136 (61.7)	59.5–64.0	0.4018	F
	Yes	164 (8.2)	107 (65.2)	57.4–72.5		
	Unknown	4 (0.2)	4 (100.0)	ND		
History of thyroid disease	No	1843 (91.8)	1130 (61.3)	59.0–63.5	**0.0055**	F
	Yes	153 (7.6)	111 (72.5)	64.8–79.4		
	Unknown	12 (0.6)	6 (50.0)	ND		
History of autoimmune disease	No	1943 (96.8)	1204 (62.0)	59.8–64.1	0.5190	F
	Yes	65 (3.2)	43 (66.2)	53.4–77.4		
Stage	III	222 (11.1)	150 (67.6)	61.0–73.7	0.0609	χ^2^
	IV	1672 (83.3)	1021 (61.1)	58.7–63.4		
	Other	39 (1.9)	31 (79.5)	63.5–90.7		
	Unknown	75 (3.7)	45 (60.0)	ND		
Metastasis	No	88 (4.4)	57 (64.8)	53.9–74.7	0.6547	F
	Yes	1894 (94.3)	1179 (62.2)	60.0–64.4		
	Unknown	26 (1.3)	11 (42.3)	ND		
Treatment line	First	691 (34.4)	415 (60.1)	56.3–63.7	0.1783	W
	Second/later line	1187 (59.1)	750 (63.2)	60.4–65.9		
	Unknown	130 (6.5)	82 (63.1)	ND		
Recent use of molecular‐targeted drug	No	1918 (95.5)	1207 (62.9)	60.7–65.1	**0.0005**	F
	Yes	90 (4.5)	40 (44.4)	34.0–55.3		
Concomitant radiotherapy	No	1474 (73.4)	916 (62.1)	59.6–64.6	0.7684	F
	Yes	387 (19.3)	244 (63.0)	58.0–67.9		
	Unknown	147 (7.3)	87 (59.2)	ND		
Number of doses	1–4	722 (36.0)	355 (49.2)	45.5–52.9	**<0.0001**	W
	5–8	430 (21.4)	292 (67.9)	63.3–72.3		
	9–12	282 (14.0)	205 (72.7)	67.1–77.8		
	13–16	216 (10.8)	166 (76.9)	70.6–82.3		
	17–20	246 (12.3)	156 (63.4)	57.1–69.4		
	21–24	55 (2.7)	38 (69.1)	55.2–80.9		
	≥25	57 (2.8)	35 (61.4)	47.6–74.0		

*Note*: See Table S4 for results according to additional baseline factors (body weight, concomitant vaccine, concomitant immunotherapy, and laboratory tests).

Abbreviations: BMI, body mass index; CI, confidence interval; ECOG PS, Eastern Cooperative Oncology Group performance status; F, Fisher's exact test; ND, not determined; TRAE, treatment‐related adverse event; W, Wilcoxon rank‐sum test.

^†^ *p*‐values in bold are significant at <0.05.

When patients were divided by PS, the frequency of TRAE was higher in those with PS of 0–1 (64.6%) than in those with PS ≥2 (45.7%) (*p* < 0.0001) (Table 3). When PS was also cross‐tabulated by the number of doses of nivolumab, the percentage of patients who received only 1–4 doses was higher in patients with PS ≥2 than in patients with PS of 0–1 (Table S5). Overall nivolumab exposure was lower in patients with poor PS.

This PMS included patients with a history of hepatic disease, lung disease, thyroid disease, renal disease, and autoimmune disease (Table 1). The frequency of TRAE was slightly higher in patients with a history of these diseases compared with that in patients without (65.0% vs 56.1%; *p* = 0.0002). This was also apparent in patients with a history of thyroid disease (72.5% vs 61.3%; *p* = 0.0055) (Table 3), with a higher frequency of thyroid function‐related endocrine disorders, in particular (hypothyroidism, 25.5% vs 13.1%; hyperthyroidism, 9.2% vs 4.0%; thyroid disorder, 3.3% vs 0.7%) (Table S6).

#### 3.3.3 TRAE of special interest

Treatment‐related adverse events of special interest that occurred in ≥5% of patients were thyroid dysfunction in 499 patients (24.9%), hepatic dysfunction in 413 patients (20.6%), infusion reactions in 229 patients (11.4%), colitis/severe diarrhea in 127 patients (6.3%), and ILD in 101 patients (5.0%) (Table S7). These TRAE occurred throughout the observation period (Table S8, Figure S1).

Among TRAE of special interest (any grade), the incidence of ILD and hepatic dysfunction in this PMS was ≥1% higher than the corresponding values in the Japanese and global clinical studies (Table 4). Furthermore, several types of TRAE, which were not reported in the previous studies (ONO‐4538‐02, ONO‐4538‐08, CheckMate 066, and CheckMate 037),^7^, ^8^, ^9^, ^10^ were observed in this PMS. These included myasthenia gravis/myocarditis/myositis/rhabdomyolysis (18 patients, 0.9%), venous thromboembolism (five patients, 0.2%), immune thrombocytopenic purpura (three patients, 0.1%), and encephalitis (one patient, 0.0%) (Table 4).

**TABLE 4:** Treatment‐related adverse events of special interest

Category	This surveillance (n = 2008)		Japanese studies (n = 59)^a^		Global studies (n = 474)^b^	
	All	Grade ≥3	All	Grade ≥3	All	Grade ≥3
ILD	101 (5.0)	37 (1.8)	1 (1.7)	0	11 (2.3)	0
Myasthenia gravis/myocarditis/myositis/rhabdomyolysis	18 (0.9)	11 (0.5)	0	0	0	0
Colitis/severe diarrhea	127 (6.3)	35 (1.7)	6 (10.2)	2 (3.4)	78 (16.5)	6 (1.3)
T1DM	19 (0.9)	17 (0.8)	0	0	1 (0.2)	1 (0.2)
Hepatic dysfunction	413 (20.6)	107 (5.3)	8 (13.6)	4 (6.8)	33 (7.0)	9 (1.9)
Thyroid dysfunction	499 (24.9)	12 (0.6)	17 (28.8)	0	36 (7.6)	1 (0.2)
Neurological disorder	10 (0.5)	2 (0.1)	2 (3.4)	0	9 (1.9)	3 (0.6)
Renal disorder	22 (1.1)	6 (0.3)	3 (5.1)	1 (1.7)	9 (1.9)	3 (0.6)
Adrenal disorder	24 (1.2)	13 (0.6)	0	0	1 (0.2)	0
Encephalitis	1 (0.0)	1 (0.0)	0	0	0	0
Severe skin disorder	19 (0.9)	12 (0.6)	1 (1.7)	0	4 (0.8)	1 (0.2)
Venous thromboembolism	5 (0.2)	3 (0.1)	0	0	0	0
Infusion reactions (within 24 h)	122 (6.1)	7 (0.3)	–	–	–	–
Infusion reactions	229 (11.4)	23 (1.1)	19 (32.2)	0	169 (35.7)	5 (1.1)
Immune thrombocytopenic purpura	3 (0.1)	3 (0.1)	0	0	0	0
Cardiac disorder	17 (0.8)	7 (0.3)	3 (5.1)	0	10 (2.1)	1 (0.2)

*Note*: Values are n (%).

Abbreviations: ILD, interstitial lung disease; T1DM, type 1 diabetes mellitus.

^a^ Studies ONO‐4538‐02 and ONO‐4538‐08.^7^, ^8^

^b^ Studies CheckMate 066 and CheckMate 037.^9^, ^10^

#### 3.3.4 Outcomes of TRAE of special interest

Table S8 summarizes the interventions and outcomes for thyroid dysfunction, hepatic dysfunction, colitis/severe diarrhea, and ILD. Over half of the cases resolved spontaneously or following appropriate interventions. Similar recoveries were reported for the TRAE myasthenia gravis/myocarditis/myositis/rhabdomyolysis (10/18 patients), venous thromboembolism (4/5 patients), immune thrombocytopenic purpura (2/3 patients), and encephalitis (1/1 patients). Some cases of thyroid dysfunction (37.9%) were irreversible.

#### 3.3.5 Risk factors for ILD or hepatic dysfunction

History of ILD and male sex were risk factors for nivolumab‐induced ILD in univariate and multivariable analyses (Table S9). Age (<75 years) was the risk factor for nivolumab‐induced hepatic dysfunction in univariate and multivariable analyses (Table S10).

### 3.4 Overall survival

**FIGURE 3:**
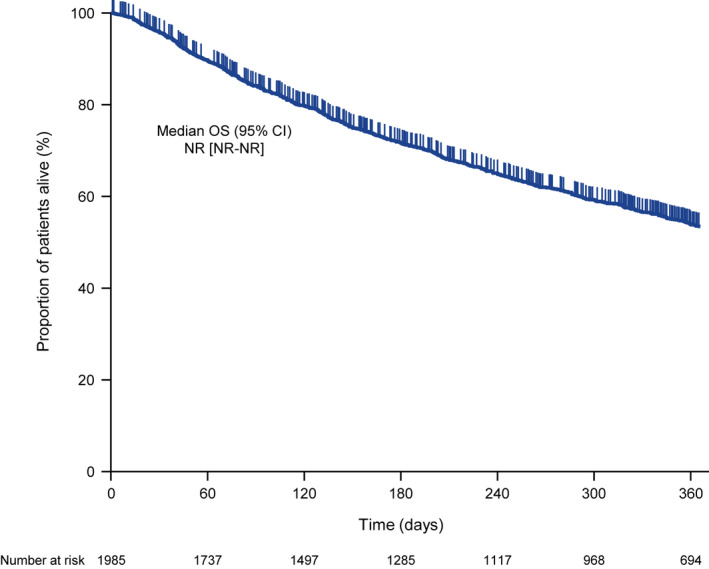
Kaplan–Meier curve of overall survival. CI, confidence interval; NR, not reached

Kaplan–Meier curves of OS are shown in Figure 3. At 12 months, median OS was not reached.

## 4 DISCUSSION

This PMS registered just over 2000 Japanese melanoma patients who started nivolumab treatment within 2.5 years of its approval in Japan. In Japan, there are approximately 2000 new melanoma patients annually of which approximately 30% (IIIA, 5.7%; IIIB, 9.7%; IIIC, 8.7%; IV, 6.2%) are stage III–IV melanoma.^1^, ^2^ Thus, it is estimated that approximately 600 patients are diagnosed with stage III–IV melanoma per year. Although not all patients with stage III–IV melanoma are candidates for systemic therapy, it is expected that most melanoma patients who had received medical therapy during this period were enrolled in this PMS. Therefore, the results of this PMS provide not only important data regarding the use of nivolumab in Japan, but also important insights regarding Japanese melanoma patients for whom drug therapy is indicated. For example, one‐third of the patients in this study had mucosal melanoma although it occupied only 10% in Japanese melanoma patients in previous reports,^2^ which suggests poorer prognosis of mucosal melanoma. The primary aim of this PMS was to evaluate the safety of nivolumab in the real‐world setting. The results showed that the frequency of TRAE in this study (62.1%) was not higher than that reported in the Japanese or global pre‐authorization phase II/III studies (75.0%).^7^, ^8^, ^9^, ^10^

Some TRAE were reported in this PMS, including myasthenia gravis/myocarditis/myositis/rhabdomyolysis, venous thromboembolism, immune thrombocytopenic purpura, and encephalitis, which were not reported in clinical studies (ONO‐4538‐02, ONO‐4538‐08, CheckMate 066, and CheckMate 037).^7^, ^8^, ^9^, ^10^ The majority of these TRAE resolved with appropriate interventions. In addition, these TRAE have been listed for call to attention in the current Risk Management Plan. Some of these TRAE and other TRAE of special interest were reported as grade 5 events (Table S7). Moreover, some TRAE of special interest were reported throughout the 1‐year observation period. Therefore, caution is necessary throughout nivolumab treatment in order to detect these TRAE and consider appropriate interventions.

Thyroid dysfunction, the most common class of TRAE of special interest, occurred in one‐quarter of patients in this study (24.9%), with a frequency similar to that in the Japanese phase II studies (17/59 patients, 28.8%), but was higher than that in the global phase III studies (36/474 patients, 7.6%) (Table 4). The incidence of grade ≥3 thyroid dysfunction was 0.6% in this PMS, 0% in the Japanese phase II studies, and 0.2% in the global phase III studies, showing a similar incidence. In a PMS of nivolumab for head and neck cancer,^13^ a global phase III study of nivolumab for head and neck cancer (CheckMate 141),^14^ and Japanese patients in CheckMate 141,^15^ thyroid dysfunction occurred in 10.2%, 7.2%, and 11.1%, respectively,^13^ which suggests that the incidence of thyroid dysfunction is not particularly high in Japanese patients. Similar results were reported in patients with non‐small cell lung cancer.^16^ The reason for the higher incidence of thyroid dysfunction among Japanese patients with melanoma is unclear. Further studies are needed, including data on other cancers.

Interstitial lung disease was more frequent in this study (5.0% of patients) than in the Japanese phase II studies (1/59 patients, 1.7%) and global phase III studies (11/474 patients, 2.3%) (Table 4). Pneumonitis and other lung disorders are well recognized among patients treated with immune checkpoint inhibitors.^17^ Japanese individuals tend to develop ILD more frequently (4% vs 0.2% for the rest of the world),^18^, ^19^ possibly due to genetic factors,^20^ which may explain the higher frequency in this study than in the global phase III studies.^9^, ^10^ The low frequency of ILD in the Japanese phase II studies might be explained by the exclusion of patients with coexisting or history of ILD or pulmonary fibrosis.^8^ It was previously suggested that pre‐existing lung abnormalities are risk factors for ILD in patients treated with immune checkpoint inhibitors.^21^ In this PMS, a history of ILD (hazard ratio [HR], 4.67; 95% confidence interval [CI], 2.14–10.19) and sex (male) (HR, 2.26; 95% CI, 1.47–3.47) were identified as risk factors for ILD in the multivariable analysis. Owing to the risk and potential severity of ILD, clinicians should be aware of this TRAE, and consider regular imaging and interventions aimed at preventing the exacerbation of lung disorders.^22^, ^23^

Hepatic dysfunction was more frequent in this study (20.6% of patients) than in the Japanese phase II studies (8/59 patients, 13.6%) and global phase III studies (33/474 patients, 7.0%) (Table 4). In this PMS, we also identified age <75 years (≥75 vs <75 years; HR, 0.80; 95% CI, 0.64–0.99) as a risk factor for hepatic dysfunction in the multivariable analysis. The incidence of hepatic dysfunction was expected to be higher in the elderly patients due to decreased hepatic function, but the opposite was true. The proportion of patients aged <75 years was 84.0% (351/418) in CheckMate 066^10^ and 68.2% (1370/2008) in this PMS. The difference in the proportion of patients aged <75 years does not explain the higher frequency of hepatic dysfunction in this PMS. The proportion of patients with poor baseline conditions is often higher in PMS than in clinical studies. In clinical trials, common exclusion criteria are PS ≥2, poor baseline hepatic function (elevated aspartate aminotransferase, alanine aminotransferase, and total bilirubin), and hepatitis B or C infection.^10^, ^14^ The differing eligibility between clinical studies and PMS may have influenced the difference in the frequency of hepatic dysfunction.

The results of this PMS should be interpreted with caution, considering its limitations, including the absence of a control group, the 1‐year observation period, and absence of central assessment of TRAE.

In conclusion, these results suggested that there was no concern requiring additional precautions for the safety of nivolumab in Japanese patients with malignant melanoma other than the safety information in the Risk Management Plan.

## CONFLICT OF INTEREST

H.U. has received consultancy fees from Ono Pharmaceutical; research grants from Ono Pharmaceutical, Maruho, Taiho Pharmaceutical, and Torii Pharmaceutical; and speakers' fees from Ono Pharmaceutical and Novartis. T.T. has received consultancy fees from Ono Pharmaceutical and Bristol‐Myers Squibb. Y.K. has received research grants from Ono Pharmaceutical; speakers' fees from Ono Pharmaceutical, Novartis, AstraZeneca, Otsuka Pharmaceutical, Janssen, Merck Bio, Maruho, and Bristol‐Myers Squibb; and conference/travel support from Bristol‐Myers Squibb and Merck. A.A. is an employee of and holds shares in Ono Pharmaceutical. T.S. is an employee of and holds shares in Ono Pharmaceutical. N.Y. has received research grants from Ono Pharmaceutical, Bristol‐Myers Squibb, Novartis, and Amgen; and speakers' fees from Ono Pharmaceutical, Bristol‐Myers Squibb, Novartis, and MSD. None of the authors are members of the Editorial Board for the *Journal of Dermatology*.

## Supporting information


Appendix S1

## Data Availability

Qualified researchers may request Ono Pharma to disclose individual patient‐level data from clinical studies through the following website: https://www.clinicalstudydatarequest.com/. For more information on Ono Pharma's Policy for the Disclosure of Clinical Study Data, please see the following website: https://www.ono.co.jp/eng/rd/policy.html.
