# Supplementary material for: Safety and effectiveness of nivolumab in Japanese patients with malignant melanoma: Final analysis of a post‐marketing surveillance
Source: J Dermatol. 2022 May 23;49(9):862–71. doi: 10.1111/1346-8138.16432 (PMC9545090; doi:10.1111/1346-8138.16432)
Supplement: Supplementary file 1 — Appendix S1 [file JDE-49-862-s001.pdf]

SUPPORTING INFORMATION

Safety and effectiveness of nivolumab in Japanese patients with malignant melanoma: Final analysis of a post-marketing surveillance

Hisashi Uhara, Tetsuya Tsuchida, Yoshio Kiyohara, Ayumi Akamatsu, Takahiko Sakamoto, Naoya Yamazaki

Contents

	Page
Table S1. Nivolumab administration	2
Table S2. Treatment-related adverse events occurring in ≥3% of patients by system organ class/preferred term	3
Table S3. Treatment-related adverse events by grade	5
Table S4. Treatment-related adverse events by patient background factors (other items)	7
Table S5. Cross-tabulation of number of doses of nivolumab, performance status, and treatment-related adverse events	9
Table S6. Treatment-related adverse events by system organ class (and preferred term for endocrine disorders) in patients with any medical history or history of thyroid disease	10
Table S7. Treatment-related adverse events of special interest	12
Table S8. Outcomes of treatment-related adverse events of special interest: thyroid dysfunction, hepatic dysfunction, colitis/severe diarrhea, and ILD	13
Table S9. Univariate and multivariable analyses of risk factors for ILD	15
Table S10. Univariate and multivariable analyses of risk factors for hepatic dysfunction	17
Figure S1. Time to onset and recovery/remission of treatment-related adverse events of special interest	18

Table S1. Nivolumab administration

		n (%)
Number of doses	1–4	722 (36.0)
	5–8	430 (21.4)
	9–12	282 (14.0)
	13–16	216 (10.8)
	17–20	246 (12.3)
	21–24	55 (2.7)
	25–	57 (2.8)
	Mean ± SD	8.9 ± 6.8
	Median (range)	7.0 (1–36)
Mean individual dose per body weight (mg/kg)	>0 to <2	14 (0.7)
	2	1458 (72.6)
	>2 to <3	103 (5.1)
	3	433 (21.6)
	>3	0
	Mean ± SD	2.24 ± 0.42
	Median (range)	2.00 (0.9–3.0)

Values are *n* (%) unless otherwise specified

SD, standard deviation

Table S2. Treatment-related adverse events occurring in ≥3% of patients by system organ class/preferred term

TRAEs by system organ class/preferred term	This study (*n* = 2008)	Prior studies[†] (*n* = 533)
Any TRAE	1247 (62.1)	400 (75.0)
Endocrine disorders	394 (19.6)	50 (9.4)
Hypothyroidism	281 (14.0)	38 (7.1)
Hyperthyroidism	88 (4.4)	13 (2.4)
Skin and subcutaneous tissue disorders	339 (16.9)	216 (40.5)
Dry skin	9 (0.4)	23 (4.3)
Leukoderma	110 (5.5)	11 (2.1)
Pruritus	84 (4.2)	102 (19.1)
Rash	70 (3.5)	68 (12.8)
Maculo-papular rash	14 (0.7)	26 (4.9)
Vitiligo	6 (0.3)	46 (8.6)
Gastrointestinal disorders	191 (9.5)	181 (34.0)
Abdominal pain	10 (0.5)	16 (3.0)
Constipation	11 (0.5)	35 (6.6)
Diarrhea	92 (4.6)	80 (15.0)
Nausea	25 (1.2)	72 (13.5)
Vomiting	12 (0.6)	27 (5.1)
General disorders and administration site conditions	164 (8.2)	207 (38.8)
Asthenia	1 (0.0)	35 (6.6)
Fatigue	14 (0.7)	129 (24.2)
Malaise	63 (3.1)	7 (1.3)
Pyrexia	74 (3.7)	30 (5.6)
Hepatobiliary disorders	162 (8.1)	5 (0.9)
Hepatic function abnormal	126 (6.3)	1 (0.2)
Respiratory, thoracic and mediastinal disorders	141 (7.0)	51 (9.6)
Cough	10 (0.5)	16 (3.0)
Dyspnea	6 (0.3)	17 (3.2)
Interstitial lung disease	79 (3.9)	2 (0.4)
Metabolism and nutrition disorders	86 (4.3)	54 (10.1)
Decreased appetite	40 (2.0)	32 (6.0)
Nervous system disorders	78 (3.9)	65 (12.2)
Dysgeusia	15 (0.7)	18 (3.4)
Headache	3 (0.1)	19 (3.6)

Musculoskeletal and connective tissue disorders	65 (3.2)	69 (12.9)
Arthralgia	18 (0.9)	28 (5.3)
Myalgia	10 (0.5)	20 (3.8)
Infections and infestations	62 (3.1)	27 (5.1)
Blood and lymphatic system disorders	43 (2.1)	37 (6.9)
Anemia	14 (0.7)	26 (4.9)
Eye disorders	31 (1.5)	23 (4.3)
Angiopathy	17 (0.8)	21 (3.9)
Laboratory test	446 (22.2)	93 (17.4)
Alanine aminotransferase increased	139 (6.9)	22 (4.1)
Aspartate aminotransferase increased	170 (8.5)	22 (4.1)
Blood thyroid stimulating hormone increased	64 (3.2)	10 (1.9)
γ-glutamyl transferase increased	110 (5.5)	7 (1.3)
Blood alkaline phosphatase increased	121 (6.0)	12 (2.3)

Values are *n* (%)

†Studies ONO-4538-02, ONO-4538-08, CheckMate 066, and CheckMate 037[1-4]

TRAE, treatment-related adverse event

1 Yamazaki N, Kiyohara Y, Uhara H et al. Cytokine biomarkers to predict antitumor responses to nivolumab suggested in a phase 2 study for advanced melanoma. *Cancer Sci* 2017; **108**(5): 1022-1031. DOI: 10.1111/cas.13226.

2 Yamazaki N, Kiyohara Y, Uhara H et al. Efficacy and safety of nivolumab in Japanese patients with previously untreated advanced melanoma: A phase II study. *Cancer Sci* 2017; **108**(6): 1223-1230. DOI: 10.1111/cas.13241.

3 Weber JS, D'Angelo SP, Minor D et al. Nivolumab versus chemotherapy in patients with advanced melanoma who progressed after anti-CTLA-4 treatment (CheckMate 037): a randomised, controlled, open-label, phase 3 trial. *Lancet Oncol* 2015; **16**(4): 375-384. DOI: 10.1016/s1470-2045(15)70076-8.

4 Robert C, Long GV, Brady B et al. Nivolumab in previously untreated melanoma without BRAF mutation. *N Engl J Med* 2015; **372**(4): 320-330. DOI: 10.1056/NEJMoa1412082.

Table S3. Treatment-related adverse events by grade

System organ class	Grade 1	Grade 2	Grade 3	Grade 4	Grade 5	Unknown	Total
Infections and infestations	4 (0.2)	4 (0.2)	15 (0.7)	7 (0.3)	8 (0.4)	8 (0.4)	46 (2.3)
Neoplasms benign, malignant and unspecified (incl. cysts and polyps)	2 (0.1)	1 (0.0)	3 (0.1)	3 (0.1)	1 (0.0)	0	10 (0.5)
Blood and lymphatic system disorders	1 (0.0)	4 (0.2)	12 (0.6)	9 (0.4)	4 (0.2)	2 (0.1)	32 (1.6)
Immune system disorders	0	0	3 (0.1)	1 (0.0)	0	0	4 (0.2)
Endocrine disorders	196 (9.8)	125 (6.2)	29 (1.4)	7 (0.3)	0	9 (0.4)	366 (18.2)
Metabolism and nutrition disorders	26 (1.3)	14 (0.7)	17 (0.8)	18 (0.9)	1 (0.0)	7 (0.3)	83 (4.1)
Mental disorders	1 (0.0)	0	1 (0.0)	1 (0.0)	0	0	3 (0.1)
Nervous system disorder	14 (0.7)	6 (0.3)	10 (0.5)	6 (0.3)	2 (0.1)	2 (0.1)	40 (2.0)
Eye disorders	2 (0.1)	8 (0.4)	6 (0.3)	0	0	0	16 (0.8)
Cardiac disorders	0	0	2 (0.1)	2 (0.1)	3 (0.1)	1 (0.0)	8 (0.4)
Angiopathy	1 (0.0)	2 (0.1)	6 (0.3)	1 (0.0)	1 (0.0)	2 (0.1)	13 (0.6)
Respiratory, thoracic and mediastinal disorders	38 (1.9)	33 (1.6)	33 (1.6)	4 (0.2)	11 (0.5)	3 (0.1)	122 (6.1)
Gastrointestinal disorder	55 (2.7)	56 (2.8)	40 (2.0)	6 (0.3)	3 (0.1)	15 (0.7)	175 (8.7)
Hepatobiliary disorders	63 (3.1)	26 (1.3)	34 (1.7)	14 (0.7)	6 (0.3)	13 (0.6)	156 (7.8)
Skin and subcutaneous tissue disorders	180 (9.0)	49 (2.4)	29 (1.4)	3 (0.1)	0	28 (1.4)	289 (14.4)
Musculoskeletal and connective tissue disorders	11 (0.5)	15 (0.7)	14 (0.7)	3 (0.1)	2 (0.1)	6 (0.3)	51 (2.5)
Renal and urinary disorders	4 (0.2)	6 (0.3)	6 (0.3)	0	3 (0.1)	0	19 (0.9)

General disorders and administration site conditions	65 (3.2)	34 (1.7)	7 (0.3)	0	11 (0.5)	19 (0.9)	136 (6.8)
Laboratory test	242 (12.1)	54 (2.7)	72 (3.6)	20 (1.0)	0	19 (0.9)	407 (20.3)
Injury, poisoning and procedural complications	22 (1.1)	18 (0.9)	9 (0.4)	0	1 (0.0)	2 (0.1)	52 (2.6)

Values are *n* (%)

Table S4. Treatment-related adverse events by patient background factors (other items)

Baseline stratification factors		Patients	Patients experiencing any TRAEs			
		n (%)	*n* (%)	95% CI of incidence rate	$P^{\dagger}$	Test
All		2008	1247 (62.1)	59.9–64.2		
Body weight (kg)	<50	576 (28.7)	331 (57.5)	53.3–61.5	**0.0181**	W
	50 to <60	635 (31.6)	404 (63.6)	59.7–67.4		
	60 to <70	499 (24.9)	321 (64.3)	60.0–68.5		
	70 to <80	216 (10.8)	138 (63.9)	57.1–70.3		
	≥80	72 (3.6)	47 (65.3)	53.1–76.1		
	Unknown	10 (0.5)	6 (60.0)	ND		
Concomitant vaccine	No	1978 (98.5)	1222 (61.8)	59.6–63.9	**0.0206**	F
	Yes	29 (1.4)	24 (82.8)	64.2–94.2		
	Unknown	1 (0.0)	1 (100.0)	ND		
Concomitant immunotherapy	No	1776 (88.4)	1099 (61.9)	59.6–64.1	**0.0097**	F
	Yes	60 (3.0)	47 (78.3)	65.8–87.9		
	Unknown	172 (8.6)	101 (58.7)	ND		
AST (U/L)	<33	1597 (79.5)	1004 (62.9)	60.4–65.2	0.1709	W
	≥33	328 (16.3)	193 (58.8)	53.3–64.2		
	Unknown	83 (4.1)	50 (60.2)	ND		
ALT (U/L)	<42	1740 (86.7)	1090 (62.6)	60.3–64.9	0.1625	W
	≥42	183 (9.1)	105 (57.4)	49.9–64.6		
	Unknown	85 (4.2)	52 (61.2)	ND		
γ-GTP (U/L)	<70	1351 (67.3)	865 (64.0)	61.4–66.6	**0.0354**	W
	≥70	367 (18.3)	213 (58.0)	52.8–63.1		
	Unknown	290 (14.4)	169 (58.3)	ND		
ALP (U/L)	<359	1429 (71.2)	908 (63.5)	61.0–66.0	0.1308	W
	≥359	312 (15.5)	184 (59.0)	53.3–64.5		
	Unknown	267 (13.3)	155 (58.1)	ND		
Total bilirubin (mg/dL)	<1.0	1615 (80.4)	1010 (62.5)	60.1–64.9	0.3475	W
	≥1.0	193 (9.6)	114 (59.1)	51.8–66.1		
	Unknown	200 (10.0)	123 (61.5)	ND		

TSH (μIU/mL)	<0.45	57 (2.8)	38 (66.7)	52.9–78.6	0.5423‡	W
	≥0.45 to <4.5	1458 (72.6)	914 (62.7)	60.1–65.2	0.1364§	W
	≥4.5	186 (9.3)	127 (68.3)	61.1–74.9		
	Unknown	307 (15.3)	168 (54.7)	ND		
FT_3 (pg/mL)	<1.7	74 (3.7)	32 (43.2)	31.8–55.3	**0.0004¶**	W
	≥1.7 to <4.0	1456 (72.5)	926 (63.6)	61.1–66.1	0.4903††	W
	≥4.0	6 (0.3)	3 (50.0)	11.8–88.2		
	Unknown	472 (23.5)	286 (60.6)	ND		
FT_4 (ng/dL)	<0.7	24 (1.2)	13 (54.2)	32.8–74.4	0.3452‡‡	W
	≥0.7 to <1.5	1560 (77.7)	991 (63.5)	61.1–65.9	0.1896§§	W
	≥1.5	105 (5.2)	60 (57.1)	47.1–66.8		
	Unknown	319 (15.9)	183 (57.4)	ND		

†*P*-values in bold are significant at <0.05

‡For ≥0.45 to <4.5 μIU/mL vs <0.45 μIU/mL

§For ≥0.45 to <4.5 μIU/mL vs ≥4.5 μIU/mL

¶For ≥1.7 to <4.0 pg/mL vs <1.7 pg/mL

††For ≥1.7 to <4.0 pg/mL vs ≥4.0 pg/mL

‡‡For ≥0.7 to <1.5 ng/dL vs <0.7 ng/dL

§§For ≥0.7 to <1.5 ng/dL vs ≥1.5 ng/dL

TRAE, treatment-related adverse event; CI, confidence interval; W, Wilcoxon rank-sum test; ND, not determined; F, Fisher's exact test; AST, aspartate aminotransferase; ALT, alanine aminotransferase; γ-GTP, γ-glutamyl transferase; ALP, alkaline phosphatase; TSH, thyroid-stimulating hormone; FT_3, free triiodothyronine; FT_4, free thyroxine

Table S5. Cross-tabulation of number of doses of nivolumab, performance status, and treatment-related adverse events

	All patients		PS 0–1		PS 2–4	
	**Patients**	**TRAEs**	**Patients**	**TRAEs**	**Patients**	**TRAEs**
Patients	2008 (100.0)	1247 (62.1)	1738 (100.0)	1123 (64.6)	269 (100.0)	123 (45.7)
Number of doses of nivolumab						
1–4	722 (36.0)	355 (49.2)	517 (29.7)	274 (53.0)	205 (76.2)	81 (39.5)
5–8	430 (21.4)	292 (67.9)	393 (22.6)	269 (68.4)	36 (13.4)	22 (61.1)
9–12	282 (14.0)	205 (72.7)	274 (15.8)	201 (73.4)	8 (3.0)	4 (50.0)
13–16	216 (10.8)	166 (76.9)	205 (11.8)	157 (76.6)	11 (4.1)	9 (81.8)
17–20	246 (12.3)	156 (63.4)	240 (13.8)	152 (63.3)	6 (2.2)	4 (66.7)
21–24	55 (2.7)	38 (69.1)	54 (3.1)	37 (68.5)	1 (0.4)	1 (100.0)
≥25	57 (2.8)	35 (61.4)	55 (3.2)	33 (60.0)	2 (0.7)	2 (100.0)

Values are *n* (%)

PS, performance status; TRAE, treatment-related adverse event

Table S6. Treatment-related adverse events by system organ class (and preferred term for endocrine disorders) in patients with any medical history or history of thyroid disease

	Any medical history		History of thyroid disease	
	No	Yes	No	Yes
N	635	1362	1843	153
Any TRAE	356 (56.1)	885 (65.0)	1130 (61.3)	111 (72.5)
Infections and infestations	11 (1.7)	51 (3.7)	59 (3.2)	3 (2.0)
Neoplasms benign, malignant and unspecified (incl. cysts and polyps)	1 (0.2)	13 (1.0)	14 (0.8)	0
Blood and lymphatic system disorders	10 (1.6)	32 (2.3)	41 (2.2)	1 (0.7)
Immune system disorders	1 (0.2)	5 (0.4)	5 (0.3)	1 (0.7)
Endocrine disorders	112 (17.6)	279 (20.5)	340 (18.4)	52 (34.0)
Adrenal insufficiency	4 (0.6)	13 (1.0)	16 (0.9)	1 (0.7)
Adrenocortical insufficiency acute	0	1 (0.1)	1 (0.1)	0
Hyperpituitarism	0	1 (0.1)	1 (0.1)	0
Hyperthyroidism	28 (4.4)	59 (4.3)	73 (4.0)	14 (9.2)
Hypopituitarism	10 (1.6)	11 (0.8)	20 (1.1)	1 (0.7)
Hypothyroidism	78 (12.3)	201 (14.8)	241 (13.1)	39 (25.5)
Primary hypothyroidism	0	4 (0.3)	3 (0.2)	1 (0.7)
Secondary adrenocortical insufficiency	3 (0.5)	3 (0.2)	5 (0.3)	1 (0.7)
Thyroid disorder	2 (0.3)	16 (1.2)	13 (0.7)	5 (3.3)
Thyroiditis	7 (1.1)	10 (0.7)	13 (0.7)	4 (2.6)
Thyroiditis chronic	0	5 (0.4)	4 (0.2)	1 (0.7)
Autoimmune thyroiditis	3 (0.5)	1 (0.1)	4 (0.2)	0
Inappropriate ADH secretion	0	1 (0.1)	1 (0.1)	0
Hypophysitis	3 (0.5)	11 (0.8)	14 (0.8)	0
ACTH deficiency	5 (0.8)	12 (0.9)	15 (0.8)	2 (1.3)
Silent thyroiditis	1 (0.2)	4 (0.3)	4 (0.2)	1 (0.7)
Metabolism and nutrition disorders	16 (2.5)	70 (5.1)	76 (4.1)	10 (6.5)
Mental disorder	0	5 (0.4)	4 (0.2)	1 (0.7)
Nervous system disorder	13 (2.0)	65 (4.8)	71 (3.9)	6 (3.9)
Eye disorders	8 (1.3)	23 (1.7)	28 (1.5)	3 (2.0)
Ear and labyrinth disorders	1 (0.2)	2 (0.1)	3 (0.2)	0
Cardiac disorders	4 (0.6)	13 (1.0)	15 (0.8)	2 (1.3)

Angiopathy	3 (0.5)	14 (1.0)	15 (0.8)	2 (1.3)
Respiratory, thoracic and mediastinal disorders	32 (5.0)	109 (8.0)	128 (6.9)	12 (7.8)
Gastrointestinal disorder	53 (8.3)	138 (10.1)	176 (9.5)	15 (9.8)
Hepatobiliary disorders	46 (7.2)	116 (8.5)	151 (8.2)	10 (6.5)
Skin and subcutaneous tissue disorders	99 (15.6)	239 (17.5)	316 (17.1)	23 (15.0)
Musculoskeletal and connective tissue disorders	12 (1.9)	53 (3.9)	56 (3.0)	9 (5.9)
Renal and urinary disorders	3 (0.5)	23 (1.7)	23 (1.2)	3 (2.0)
Reproductive system and breast disorders	1 (0.2)	1 (0.1)	1 (0.1)	1 (0.7)
General disorders and administration site conditions	44 (6.9)	119 (8.7)	154 (8.4)	9 (5.9)
Laboratory test	128 (20.2)	316 (23.2)	401 (21.8)	43 (28.1)
Injury, poisoning and procedural complications	15 (2.4)	39 (2.9)	50 (2.7)	3 (2.0)

Values are *n* (%)

TRAE, treatment-related adverse event; ADH, antidiuretic hormone; ACTH, adrenocorticotropic hormone

Table S7. Treatment-related adverse events of special interest

	Grade 1	Grade 2	Grade 3	Grade 4	Grade 5	Unknown	Total
ILD	33 (1.6)	29 (1.4)	25 (1.2)	3 (0.1)	9 (0.4)	2 (0.1)	101 (5.0)
Myasthenia gravis/ myocarditis/myositis/ rhabdomyolysis	1 (0.0)	4 (0.2)	5 (0.2)	3 (0.1)	3 (0.1)	2 (0.1)	18 (0.9)
Colitis/severe diarrhea	35 (1.7)	45 (2.2)	29 (1.4)	4 (0.2)	2 (0.1)	12 (0.6)	127 (6.3)
T1DM	0	2 (0.1)	4 (0.2)	13 (0.6)	0	0	19 (0.9)
Hepatic dysfunction	231 (11.5)	65 (3.2)	79 (3.9)	22 (1.1)	6 (0.3)	10 (0.5)	413 (20.6)
Thyroid dysfunction	340 (16.9)	133 (6.6)	10 (0.5)	2 (0.1)	0	14 (0.7)	499 (24.9)
Nerve disorders	5 (0.2)	3 (0.1)	2 (0.1)	0	0	0	10 (0.5)
Renal dysfunction	8 (0.4)	8 (0.4)	3 (0.1)	0	3 (0.1)	0	22 (1.1)
Adrenal dysfunction	1 (0.0)	9 (0.4)	9 (0.4)	4 (0.2)	0	1 (0.0)	24 (1.2)
Encephalitis	0	0	1 (0.0)	0	0	0	1 (0.0)
Severe skin disorders	3 (0.1)	3 (0.1)	10 (0.5)	2 (0.1)	0	1 (0.0)	19 (0.9)
Venous thromboembolism	0	2 (0.1)	2 (0.1)	0	1 (0.0)	0	5 (0.2)
Infusion reactions (within 24 h)	84 (4.2)	23 (1.1)	6 (0.3)	0	1 (0.0)	8 (0.4)	122 (6.1)
Infusion reactions	134 (6.7)	52 (2.6)	17 (0.8)	2 (0.1)	4 (0.2)	20 (1.0)	229 (11.4)
Immune thrombocytopenic purpura	0	0	0	3 (0.1)	0	0	3 (0.1)
Cardiac disorders	5 (0.2)	4 (0.2)	2 (0.1)	2 (0.1)	3 (0.1)	1 (0.0)	17 (0.8)

Values are *n* (%)

ILD, interstitial lung disease; T1DM, type 1 diabetes mellitus

Table S8. Outcomes of treatment-related adverse events of special interest: thyroid dysfunction, hepatic dysfunction, colitis/severe diarrhea, and ILD

Category	Treatment	Resolved/ resolving	Resolved with sequelae	Unresolved	Death	Unknown	Total
Thyroid dysfunction	Untreated	142 (46.6)	2 (0.7)	124 (40.7)	0	37 (12.1)	305
	Corticosteroid	2 (66.7)	0	1 (33.3)	0	0	3
	HRT	97 (57.7)	3 (1.8)	62 (36.9)	0	6 (3.6)	168
	Corticosteroid + HRT	0	0	0	0	0	0
	Other	8 (72.7)	3 (27.3)	0	0	0	11
	Unknown	4 (33.3)	0	2 (16.7)	0	6 (50.0)	12
	Total	253 (50.7)	8 (1.6)	189 (37.9)	0	49 (9.8)	499
Hepatic dysfunction	Untreated	163 (52.9)	0	127 (41.2)	2 (0.6)	16 (5.2)	308
	Corticosteroid	39 (79.6)	2 (4.1)	6 (12.2)	2 (4.1)	0	49
	Immunosuppressant	0	0	0	0	0	0
	Corticosteroid + immunosuppressant	5 (83.3)	0	1 (16.7)	0	0	6
	Other	28 (71.8)	0	7 (17.9)	2 (5.1)	2 (5.1)	39
	Unknown	3 (27.3)	0	2 (18.2)	0	6 (54.5)	11
	Total	238 (57.6)	2 (0.5)	143 (34.6)	6 (1.5)	24 (5.8)	413
Colitis/ severe diarrhea	Untreated	30 (93.8)	0	1 (3.1)	0	1 (3.1)	32
	Corticosteroid	52 (92.9)	0	3 (5.4)	1 (1.8)	0	56
	Immunosuppressant	0	0	0	0	0	0
	Corticosteroid + immunosuppressant	7 (100.0)	0	0	0	0	7
	Other	15 (75.0)	0	3 (15.0)	1 (5.0)	1 (5.0)	20
	Unknown	8 (66.7)	0	2 (16.7)	0	2 (16.7)	12

	Total	112 (88.2)	0	9 (7.1)	2 (1.6)	4 (3.1)	127
ILD	No treatment	20 (69.0)	0	9 (31.0)	0	0	29
	Corticosteroid	44 (68.8)	3 (4.7)	8 (12.5)	8 (12.5)	1 (1.6)	64
	Immunosuppressant	0	0	0	0	0	0
	Corticosteroid + immunosuppressant	1 (100.0)	0	0	0	0	1
	Other	4 (100.0)	0	0	0	0	4
	Unknown	1 (33.3)	0	1 (33.3)	1 (33.3)	0	3
	Total	70 (69.3)	3 (3.0)	18 (17.8)	9 (8.9)	1 (1.0)	101

Values are *n* (%)

HRT, hormone replacement therapy; ILD, interstitial lung disease

Table S9. Univariate and multivariable analyses of risk factors for ILD

Factor	Category	*n*	Patients with ILD, *n* (%)	Univariate† HR (95% CI)	Multivariable‡ HR (95% CI)
Sex	Female§	978	30 (3.1)	2.30 (1.50–3.52)	2.26 (1.47–3.47)
	Male	1030	71 (6.9)		
Age (years)	<75§	1370	61 (4.5)	1.45 (0.97–2.16)	-
	≥75	638	40 (6.3)		
ECOG PS	0–1§	1738	90 (5.2)	0.82 (0.44–1.54)	-
	2–4	269	11 (4.1)		
History of autoimmune disease	No§	1943	95 (4.9)	1.82 (0.81–4.08)	-
	Yes	65	6 (9.2)		
History of ILD	No§	1976	94 (4.8)	4.86 (2.23–10.62)	4.67 (2.14–10.19)
	Yes	32	7 (21.9)		
History of emphysema/COPD	No§	1972	101 (5.1)	-	-
	Yes	36	0		
History of pulmonary infection	No§	1978	98 (5.0)	2.05 (0.65–6.53)	2.12 (0.65–6.93)
	Yes	30	3 (10.0)		
Metastasis	No§	88	6 (6.8)	0.74 (0.33–1.67)	-
	Yes	1894	95 (5.0)		
Chemotherapy (previous treatment for melanoma)	No§	691	26 (3.8)	1.57 (1.00–2.45)	-
	Yes	1290	75 (5.8)		
Treatment line	First	691	26 (3.8)	0.88 (0.55–1.41)¶	-
	Second	609	33 (5.4)		
	Third/later§	578	35 (6.1)		
Recent use of molecular-targeted drug	No§	1918	96 (5.0)	1.15 (0.47–2.82)	-
	Yes	90	5 (5.6)		

†In univariate analyses, sex, history of ILD and history of pulmonary infection had HRs of <0.5 or >2; these three factors were considered as risk factors and were used as explanatory variables in the multivariable analysis

‡In multivariable analyses, the clinically relevant factors (age, ECOG PS, medical history of autoimmune disease, metastasis, treatment line, recent use of molecular targeted drugs) were individually included in models together with the three identified risk factors for ILD (sex, a medical

history of ILD and a medical history of pulmonary infection) as explanatory variables. These analyses identified sex and medical history of ILD as secondary candidate screening risk factors (data not shown). The final multivariable model comprised sex, medical history of ILD, and medical history of pulmonary infection as explanatory variables. Because the 95% CIs of the HRs for sex and history of ILD did not cross 1, these factors were considered statistically significant

[§]Reference category

[¶]For second-line vs third/later-line

ILD, interstitial lung disease; HR, hazard ratio; CI, confidence interval; ECOG PS, Eastern Cooperative Group performance status; COPD, chronic obstructive pulmonary disease

Table S10. Univariate and multivariable analyses of risk factors for hepatic dysfunction

Factor	Category	*n*	Patients with hepatic dysfunction, *n* (%)	Univariate † HR (95% CI)	Multivariable ‡ HR (95% CI)
Sex	Female§	978	186 (19.0)	1.20 (0.99–1.46)	-
	Male	1030	227 (22.0)		
Age (years)	<75§	1370	301 (22.0)	0.79 (0.63–0.98)	0.80 (0.64–0.99)
	≥75	638	112 (17.6)		
ECOG PS	0–1§	1738	360 (20.7)	1.00 (0.73–1.36)	-
	2–4	269	53 (19.7)		
History of liver disease	No§	1822	362 (19.9)	1.42 (1.05–1.91)	1.35 (0.99–1.84)
	Yes	181	50 (27.6)		
Metastasis	No§	88	16 (18.2)	1.20 (0.74–1.94)	-
	Yes	1894	395 (20.9)		
Treatment line	First	691	136 (19.7)	1.21 (0.94–1.55)¶	-
	Second	609	141 (23.2)		
	Third/later§	578	110 (19.0)		
AST (U/L)	<33§	1597	319 (20.0)	1.34 (1.04–1.72)	1.28 (0.99–1.66)
	≥33	328	81 (24.7)		
ALT (U/L)	<42§	1740	356 (20.5)	1.24 (0.89–1.72)	-
	≥42	183	44 (24.0)		
Total bilirubin (mg/dL)	<1.0§	1615	328 (20.3)	1.24 (0.91–1.69)	-
	≥1.0	193	47 (24.4)		

†In univariate analyses for hepatic dysfunction, there were no factors with a hazard ratio of <0.5 or >2. The 95% CIs of the HRs for age, history of liver disease and AST did not cross 1 and they were therefore statistically significant

‡The multivariable model comprised age, history of liver disease, and AST as explanatory variables. Because the 95% CI of the HR for age did not cross 1, this factor was considered statistically significant

§Reference category

¶For second-line vs third/later-line

HR, hazard ratio; CI, confidence interval; ECOG PS, Eastern Cooperative Group performance status; AST, aspartate aminotransferase; ALT, alanine aminotransferase

Figure S1. Time to onset and recovery/remission of treatment-related adverse events of special interest

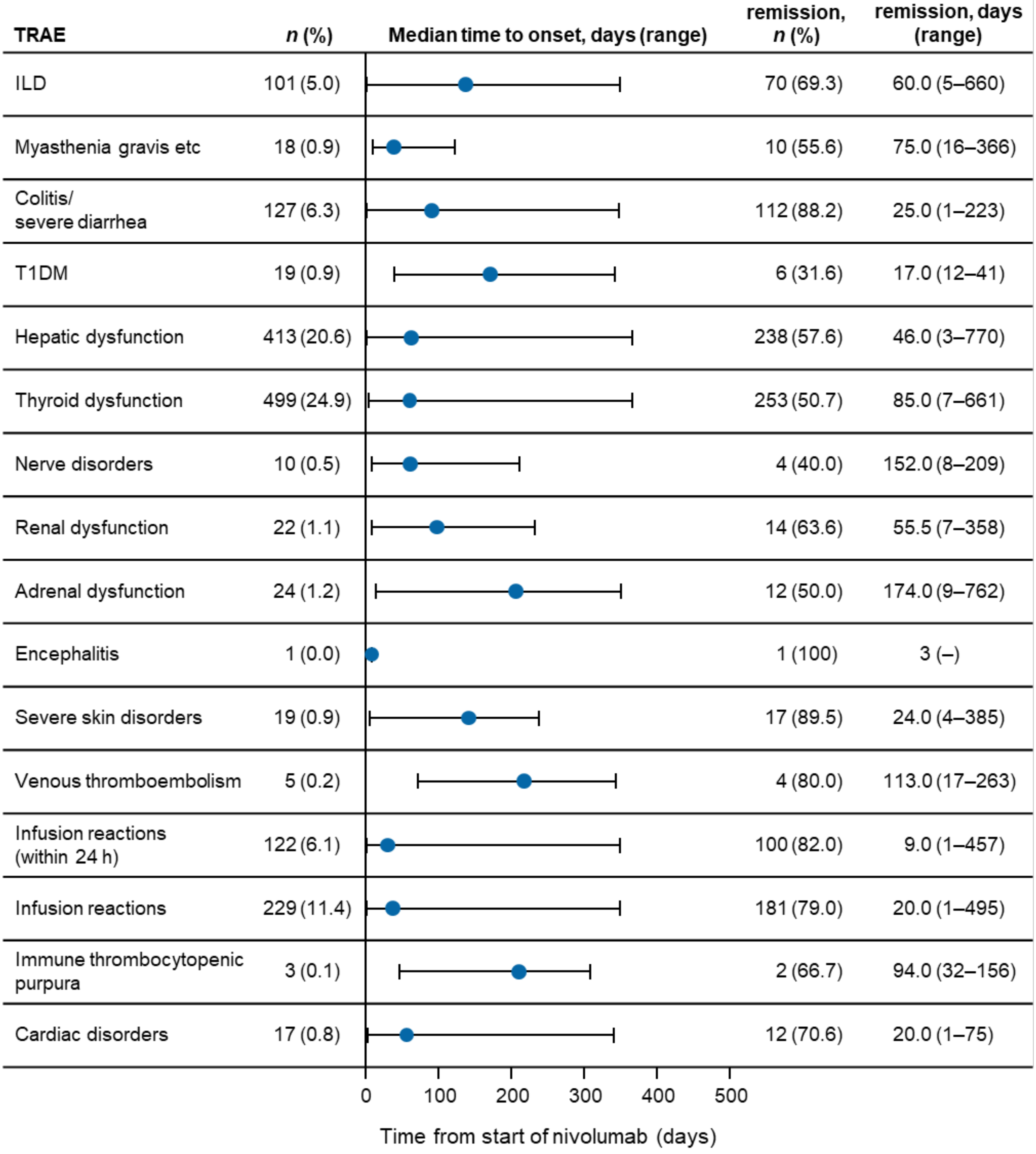

TRAE	*n* (%)	Recovery or remission, *n* (%)	Median time to recovery or remission, days (range)
ILD	101 (5.0)	70 (69.3)	60.0 (5–660)
Myasthenia gravis etc	18 (0.9)	10 (55.6)	75.0 (16–366)
Colitis/ severe diarrhea	127 (6.3)	112 (88.2)	25.0 (1–223)
T1DM	19 (0.9)	6 (31.6)	17.0 (12–41)
Hepatic dysfunction	413 (20.6)	238 (57.6)	46.0 (3–770)
Thyroid dysfunction	499 (24.9)	253 (50.7)	85.0 (7–661)
Nerve disorders	10 (0.5)	4 (40.0)	152.0 (8–209)
Renal dysfunction	22 (1.1)	14 (63.6)	55.5 (7–358)
Adrenal dysfunction	24 (1.2)	12 (50.0)	174.0 (9–762)
Encephalitis	1 (0.0)	1 (100)	3 (–)
Severe skin disorders	19 (0.9)	17 (89.5)	24.0 (4–385)
Venous thromboembolism	5 (0.2)	4 (80.0)	113.0 (17–263)
Infusion reactions (within 24 h)	122 (6.1)	100 (82.0)	9.0 (1–457)
Infusion reactions	229 (11.4)	181 (79.0)	20.0 (1–495)
Immune thrombocytopenic purpura	3 (0.1)	2 (66.7)	94.0 (32–156)
Cardiac disorders	17 (0.8)	12 (70.6)	20.0 (1–75)

ILD, interstitial lung disease; T1DM, type 1 diabetes mellitus
